# Clinical Steps for Restoration of Fractured Anterior Teeth: Color Protocol with Non-VITA Scale

**DOI:** 10.1155/2019/3982082

**Published:** 2019-05-28

**Authors:** Ubiracy Gaião, Leonardo Fernandes da Cunha, Cibele de Almeida Kintopp, André Vivan Garcia, Carla Castiglia Gonzaga, Alexandre Moro, Gisele Maria Correr

**Affiliations:** School of Health Sciences, Graduate Program in Dentistry, Universidade Positivo, Curitiba, PR, Brazil

## Abstract

Direct composite resin restorations are commonly provided because of their satisfactory esthetics and minimal wear of opposing tooth structure. Recent restorative systems may not follow the nomenclature of the classical VITA shade guide, using instead a simplified resin color system. A better understanding of these systems and their behavior regarding the incidence of light is an excellent approach to anterior restorations, especially for fractured anterior teeth. This paper demonstrates the color selection and clinical sequence for the natural reproduction of tooth structure using a resin system that does not follow the VITA classical scale.

## 1. Introduction

Natural tooth is polychromatic, presenting a great variety of colors. Artificially reproducing these intrinsic characteristics of the tooth always demands time and often excessive adjustments. Dentists should use their artistic sense to identify details and define the different hues of each tooth [1–3]. For the professional to achieve this result, the restorative system must present a series of characteristics.

Changes in direct restorative systems have addressed the growing demand of patients and professionals for esthetics and optical characteristics that match those of natural teeth. Composite resins are available in a wide variety of colors and effects that allow combinations of translucency and opacity of dental structures [3].

Recently, direct restorative systems have tended not to use the VITA shade guide scale for their available colors. Their kits feature simplified enamel and dentin color options that are selected separately. Dentin confers the basic color, or hue, of the tooth, while the enamel does not change the hue but provides increased or reduced saturation, or chroma, according to its thickness [4]. The lightness or value will result from the placement of correct thicknesses of the resin layers. Thus, the technique is simplified and the clinical time reduced.

In addition, light-polymerized stains, which are flowable composite resins with low filler content, can be used to characterize occlusal cracks and grooves, darken teeth, or mimic the chromatic characteristics of the tooth as with an incisal opaque halo. They are available in various pigments, including brown, black, blue, and white ocher, to provide a direct restoration with a more natural appearance that is harmonious to the adjacent teeth [5].

The objective of this report was to present a predictable protocol for restoring an anterior tooth using a restorative system that does not use the VITA shade guide scale.

## 2. Case Report

**Figure 1:**
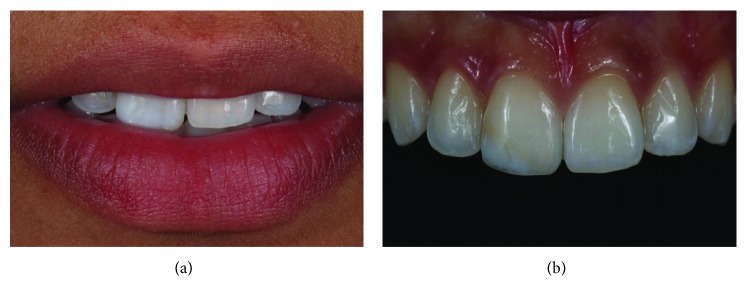
Restored right maxillary central incisor showing poor color match. The restoration of the fractured tooth extended to the middle third.

A 21-year-old woman sought care to replace the resin restoration of her fractured anterior tooth. The existing restoration had a poor color match and excess material (Figure 1). Considering the age of the patient, the possibility of reversibility of the procedure, the time, and the cost, a direct adhesive restorative system was planned to restore the tooth.

**Figure 2:**
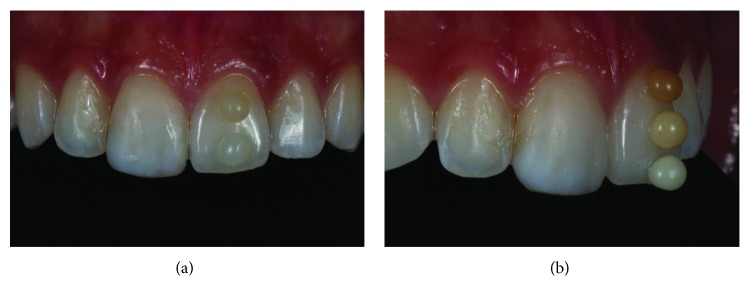
Selection of color before tooth dehydration. The two enamel colors of the system were initially tested (LE and DE) (a). Then, the three dentin colors were tested (LD, MD, and DD) (b).

After prophylaxis, the dentin and enamel color were selected using the Essentia, GC, resin system. For enamel color selection, each of the two enamel colors (light enamel and dark enamel) were placed on the tooth and polymerized (Figure 2(a)). The light enamel replicated the patient's tooth best. The dentin color was selected by applying the three dentin colors (light dentin, medium dentin, and dark dentin) on the patient's tooth and polymerizing. Light dentin was selected (Figure 2(b)).

**Figure 3:**
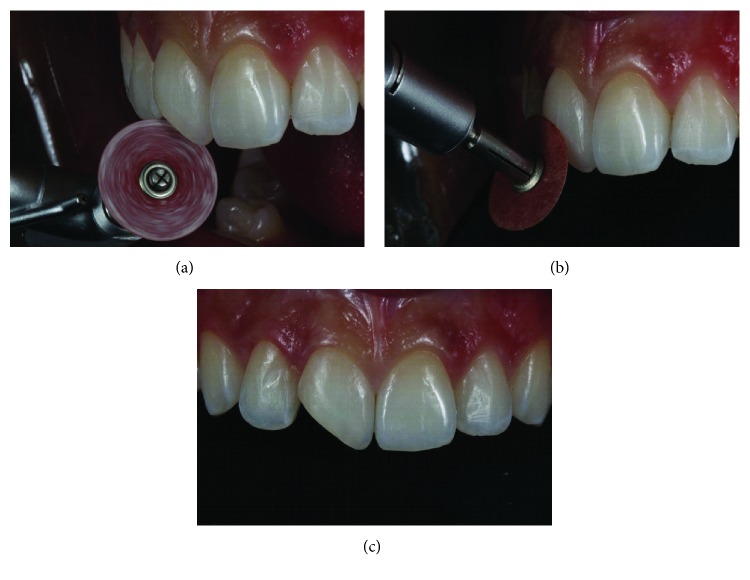
Removing the existing restoration with abrasive disks. A beveled margin was made with coarse grit disks to remove the unsupported enamel.

**Figure 4:**
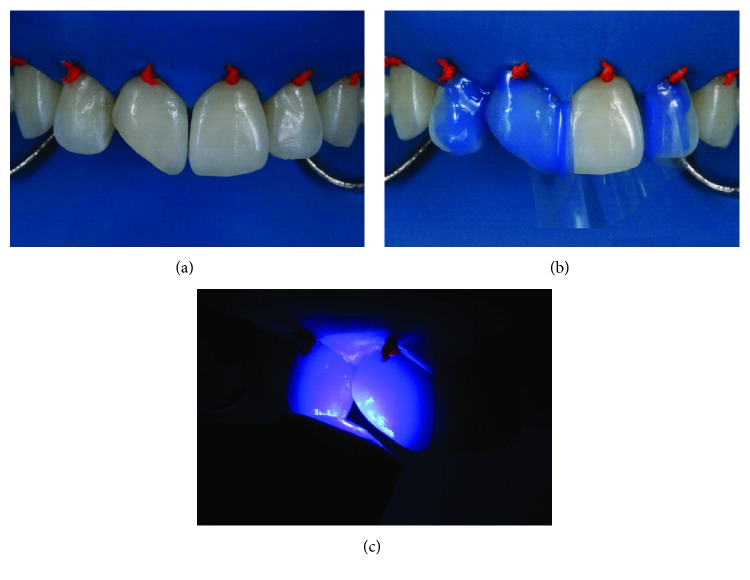
Rubber dam isolation of the operative field (a) and application of the adhesive system and polymerization according to the manufacturer's instructions (b, c).

After making a silicone putty matrix, the existing restoration was removed with abrasive disks (Sof-Lex, dark red, 3M; thick granulation). A beveled margin was made with the same disk (Figure 3). The operative field was isolated and the gingiva displaced with ligated rubber dam (Figure 4(a)). The adjacent teeth were protected with polyester tape. The enamel surface was conditioned with 37% phosphoric acid (Figure 4(b)), and the adhesive (G-BOND, GC) was then applied on the facial and lingual surfaces (Figure 4(c)) and polymerized according to the manufacturer's instructions.

**Figure 5:**
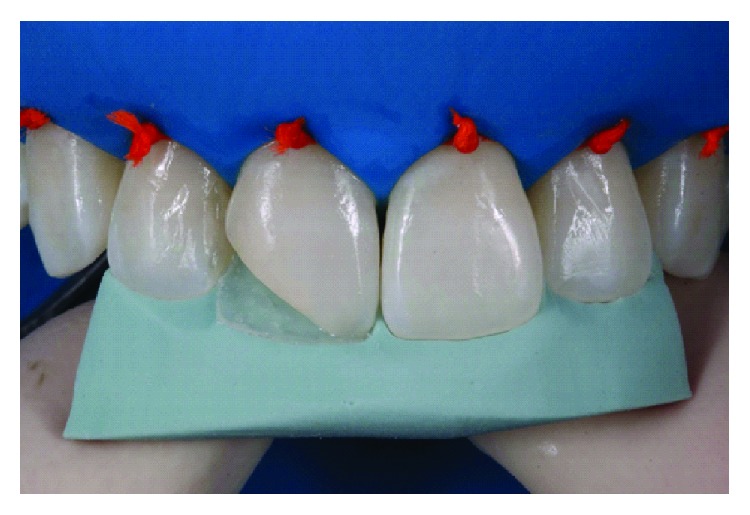
A silicone guide was used to assist the restoration of the lingual surface with the light enamel composite resin (LE: light enamel, Essentia, GC).

**Figure 6:**
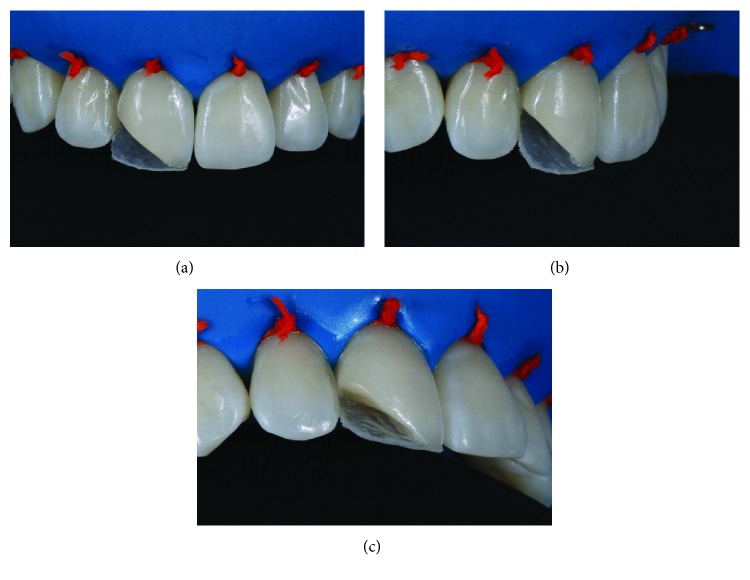
Facial (a), lateral (b), and incisal (c) views immediately after application and polymerization of the resin to form the lingual “shell” with enamel resin.

**Figure 7:**
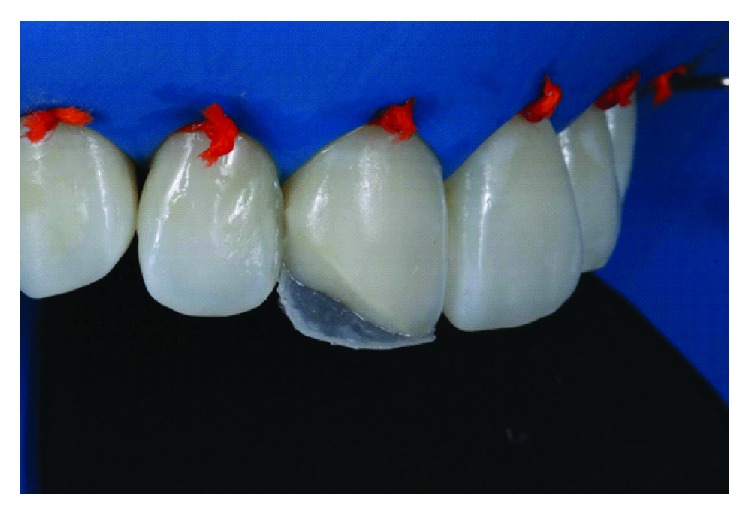
LD (light dentin; Essentia, GC) resin, corresponding to the dentin region, was then applied.

**Figure 8:**
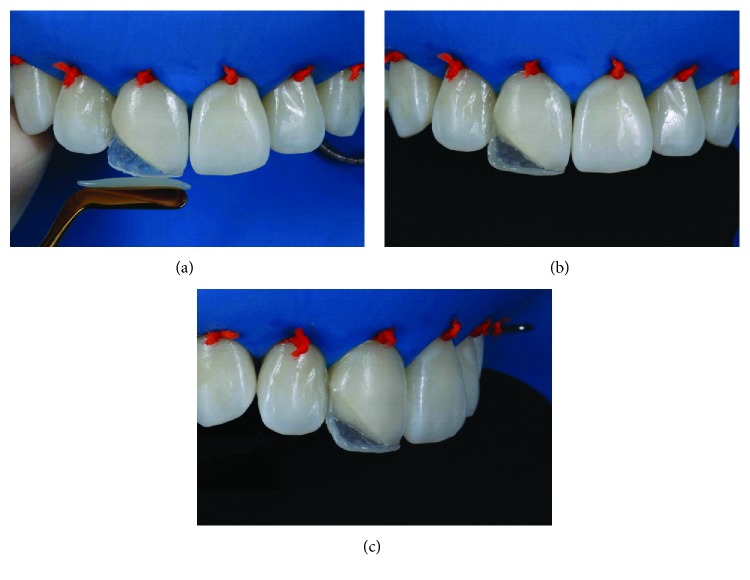
A resin with greater OM opalescence was placed to characterize the incisal area (a–c).

**Figure 9:**
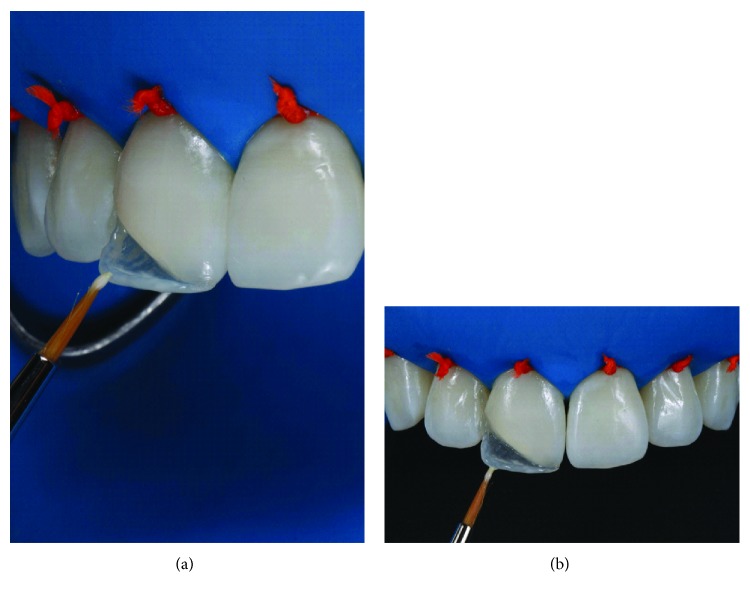
Brush application of the white stain (Essentia, white modifier) to characterize the opaque incisal halo (a, b).

**Figure 10:**
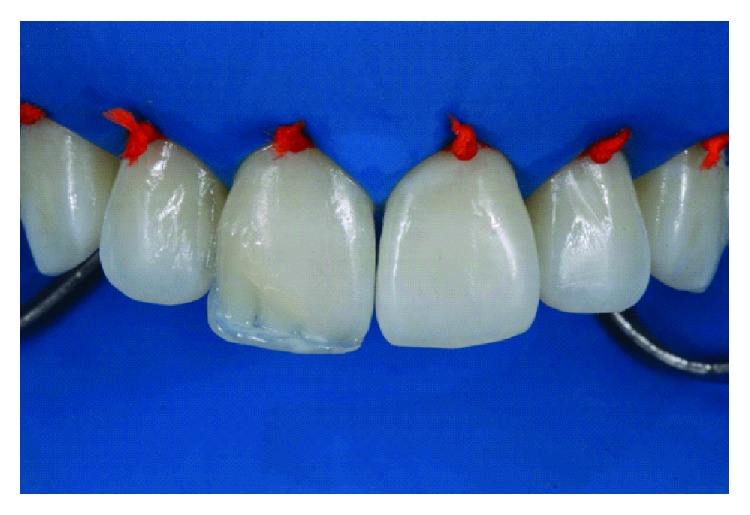
Application of LD dentin resin up to approximately half the width of the bevel. Observe the spaces between the developmental lobes, left when applying the opaque resin, in which the translucent incisal resin (OM) will be placed.

**Figure 11:**
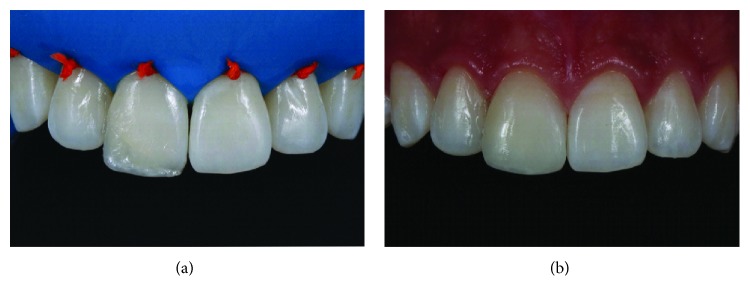
Insertion of translucent incisal resin (OM) between dentin mamelons (a). Then, the resin was applied in the LE color over the entire facial surface of the restoration to simulate the enamel (b).

The silicone matrix was positioned lingually to provide a well-contoured restoration (Figure 5(a)). Resin matching the lingual enamel was applied with the matrix in position (LE). After polymerization of this increment with the matrix in position, the lingual and incisal contour was established (Figure 6). Dentin resin was then applied to the middle third (LD), leaving room for the creation of a dentinal lobe in the incisal region (Figure 7). The incisal halo was made by using the opalescent translucent resin of the OM system (Figure 8), followed by a layer of white stain on that halo to simulate the opacity of this region (Figure 9). The dentin mamelons were made with clear resin (Figure 10), and opalescent resin was applied between the mamelons (Figure 11(a)). The enamel layer was applied to the facial surface and spread with the aid of a polyester strip and brush (Kota 4A) (Figure 11(b)).

Each increment was polymerized with an LED unit (Radii-cal, SDI) for the time recommended by the manufacturer. After removal of the rubber dam, any excess was removed, and the incisal edge adjusted.

**Figure 12:**
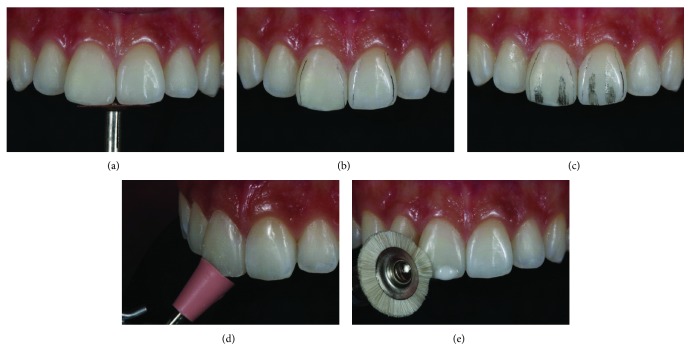
Finishing and polishing of the restoration with abrasive disks (a), fine diamond rotary instruments (b, c), rubber points (d), and a felt disk with polishing paste (e).

**Figure 13:**
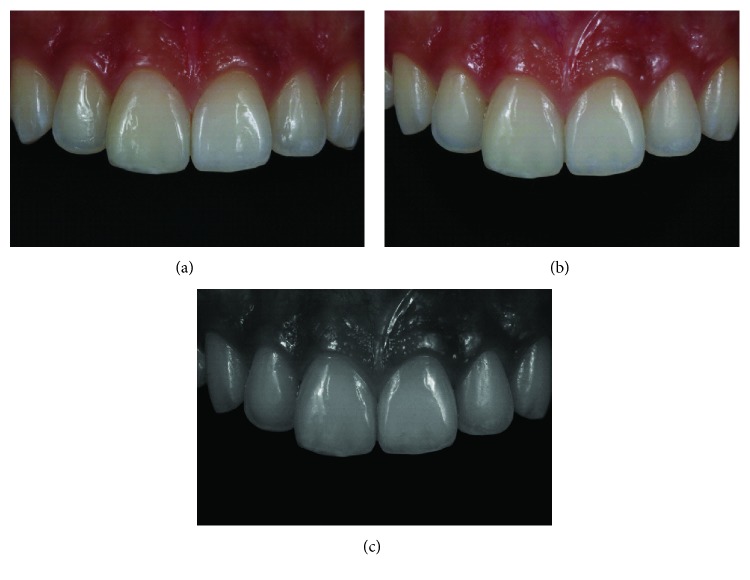
Detail of restorations after finishing and polishing procedures (a–c).

**Figure 14:**
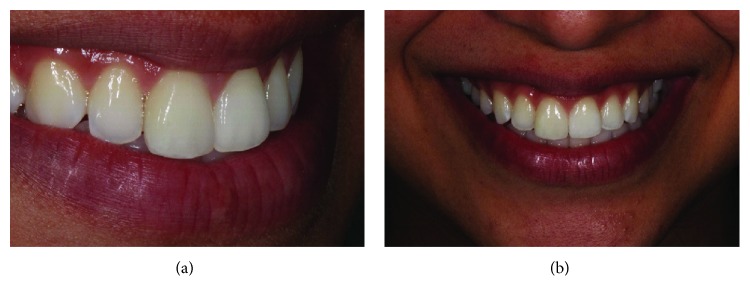
Smile view, harmony of shape, color, and function of the tooth have been restored (a, b).

In the following appointment, the restoration was finished and polished with sequential grit abrasive disks (Sof-Lex Pop-on, 3M). Rubber points and composite polishing paste applied with a felt disk were used to obtain the final gloss (Figure 12). The final restoration can be seen in Figures 13(a) and 13(b). The definitive appearance of the restored smile can be seen in Figure 14.

## 3. Discussion

Current direct adhesive restorative systems have numerous advantages such as reversibility, durability, low cost, and speed of treatment. In the patient presented, the restoration was placed in a single appointment and finished and polished in a subsequent appointment. Maximum tooth structure was conserved when the existing restoration was removed, thus preserving enamel at the margins of the preparation and favoring the adhesion and longevity of the adhesive procedure [1]. The dentist should choose the restorative system according to each situation. The system used for the present patient was recently introduced and has not been tested in many studies. However, results appear to be comparable with those of other available systems [6].

Natural teeth possess translucency, opalescence, and fluorescence, all of which must be replicated by the restorative material to achieve clinical success. Enamel translucency varies from tooth to tooth and from individual to individual. The presence or absence of color, enamel thickness, degree of translucency, and surface texture is an essential component in determining translucency [7]. The system used provides variable shades and opacities that allow the reproduction of the chromaticity and translucency/opacity of enamel and dentin. The manipulation of the thickness of the enamel and dentin increments selected without using the VITA shade guide system allows the correct characteristics of the dental structures to be reproduced in a simplified way, as demonstrated in the present treatment.

The technique of intrinsic characterization of composite resin restorations with stains is routinely used in dental offices. Several manufacturers offer stains that enable individualized and customized composite resin restorations [5]. In the treatment presented, the white stain was used. These stains should be applied carefully with a fine brush as demonstrated in this treatment to avoid excess material that can lead to an unsightly appearance or decrease in cohesive strength between the resin increments [5, 8].

The dentin resins in the restorative system used have a microhybrid composition, while the enamel resins are nanohybrid, providing increased polishability for the external layer of the restoration. The color of the composite resin is not influenced by when the restoration is polished (immediately or later). Thus, the final polishing time can be scheduled according to the preferences of the clinician [9]. In the presented patient, the definitive finishing and polishing were performed in the next session to allow rehydration of the tooth and time for the clinician to determine the need for additional resin increments.

## 4. Conclusion

The use of resins that do not use the VITA shade guide scale is easy to understand and allows a simplified application of the restorative system in a predictable, fast, and efficient way to reestablish the esthetics of anterior teeth.
